# Immunoblot for the Diagnosis of Cutaneous Leishmaniasis in French Guiana

**DOI:** 10.4269/ajtmh.19-0591

**Published:** 2021-05-03

**Authors:** Estelle Menu, Romain Blaizot, Charles Mary, Stéphane Simon, Antoine Adenis, Denis Blanchet, Coralie L’Ollivier, Stéphane Ranque, Magalie Demar

**Affiliations:** 1 Laboratoire Hospitalo-Universitaire de Parasitologie-Mycologie, Centre Hospitalier Andrée Rosemon, Cayenne, French Guiana;; 2 Laboratoire Hospitalo-Universitaire de Parasitologie-Mycologie, Institut Hospitalo-Universitaire, Méditerranée Infection, Marseille, France;; 3 Aix Marseille Université, IRD, AP-HM, IHU-Méditerranée Infection, UMR Vecteurs – Infections Tropicales et Méditerranéennes (VITROME), Marseille, France;; 4 Department of Dermatology, Andrée Rosemon Hospital, Cayenne, French Guiana;; 5 EA3593, Ecosystèmes Amazoniens et Pathologies Tropicales, University of French Guiana, Cayenne, French Guiana;; 6 Department of Internal Medicine, Andrée Rosemon Hospital, Cayenne, French Guiana

## Abstract

Cutaneous leishmaniasis (CL) is firmly established in South America. We aimed to assess the detection of IgG antibodies against 14 and/or 16 kDa antigens by immunoblot (IB) for CL serological diagnosis in French Guiana, an area where many endemic pathogens could interfere with it. This study was performed retrospectively on sera from 141 patients at the Cayenne tertiary hospital: 30 were patients with confirmed CL, 71 were diagnosed with various other endemic pathogens, 11 were diagnosed with an autoimmune disease, and 29 controls had no history of CL. Antibodies bound to the 14 and/or 16 kDa antigens in 27 of the 30 CL patients’ sera and in 39 of the 111 non-CL patients’ sera (26 from the infectious diseases group, four from the autoimmune diseases group, and nine from the dermatology department). The method tested showed a high sensitivity (90%) and a low specificity (66%), and a diagnosis odds ratio of 17.5 (95% CI [4.6–78.0]). This IB may be helpful to exclude the diagnosis of CL, prompting physicians to look for another diagnosis in the case of a negative IB.

## INTRODUCTION

New World cutaneous leishmaniasis (CL) is a vector-borne disease caused by many *Leishmania* species. This “neglected tropical disease”^1^ represents a significant public health burden in South America. According to the WHO leishmaniasis working group, the annual incidence of CL between 2004 and 2008 was estimated at 650 to 1,100 cases per year in French Guiana but is probably underestimated.^2^ This French overseas territory located in South America has an equatorial climate that is tempered by trade winds,^3^ enabling the development of *Leishmania* vectors and reservoir hosts. Five *Leishmania* species involved in human infection are sympatric in this area: *Leishmania guyanensis*, *Leishmania braziliensis*, *Leishmania amazonensis*, *Leishmania naiffi*, and *Leishmania lainsoni*.^4,5^ *Leishmania guyanensis* causes about 85% of CL cases,^6^ chiefly localized and rarely disseminated leishmaniasis,^7^ but the incidence of *L*. *braziliensis* infection, the possible causative agent of mucosal leishmaniasis, has also increased in recent years.^8^

Cutaneous leishmaniasis is difficult to diagnose because of its broad and heterogeneous clinical presentation.^9^ Conventional diagnosis of CL is based on the direct microscopic examination of Giemsa-stained smears, histopathological examination of fixed biopsies, or culture from lesion samples.^10^ However, these microscopy-based techniques display poor sensitivity because of a relatively low density and heterogeneous distribution of the parasites within clinical samples, and do not make it possible to identify the *Leishmania* species involved. Several diagnostic tools, mainly PCR-based methods, have been developed, which both increased the diagnosis sensitivity and enabled species identification.^11^

Serological diagnosis is considered to be of limited importance for CL diagnosis. Recently, ELISAs have been developed and appear to be promising.^12^ Serology has also recently demonstrated its usefulness in the diagnosis of *Leishmania* *major* (Old-World CL).^13–15^ Immunoblot (IB) techniques providing a detailed pattern of the patient’s antibody response against various leishmanial antigens^16^ are considered to be more sensitive and specific than ELISA, particularly in cases of asymptomatic visceral leishmaniasis (VL) and Old-World CL,^15^ despite the fact that the two clinical forms are very distinct. However, data on the serological diagnosis of New-World CL by IB remain scarce. Pomares et al.^15^ and Seyyedtabaei et al.^13^ demonstrated that *L. major* CL could be diagnosed using IB performed with the most specific *Leishmania infantum* antigens 14 and 16 kDa. Thus, these antigens seem to have an interspecies specificity for *Leishmania* and therefore tare of potential interest for the *Vianna* complex. This study aimed to evaluate the effectiveness of immunoblotting, targeting 14 and 16 kDa antigens, for the diagnosis of New-World CL in French Guiana.

## METHODS

### Ethical aspect.

The study was performed in accordance with the Declaration of Helsinki and used only healthcare data that are routinely used for clinical purposes in patients with all pathogens mentioned in this article.

### Patients.

Patients were included in the study if 1) at least one serum sample had been collected from them and sent to the laboratory for the diagnosis of leishmaniasis, and has been kept in the biological collection of the Cayenne Hospital laboratory (French Guiana); and 2) they complied with the case definition of the following four groups.

#### Group I.

This group included patients with a CL diagnosis that was confirmed by direct microscopical examination of lesions, positive *Leishmania* PCR, and/or culture. Species identification was made via polymerase chain reaction-restriction fragment length polymorphism^4^ and/or matrix assisted laser desorption ionization - time of flight.^17^ For each patient, demographic data; duration of symptoms; the number, distribution, and description of the skin or mucosal lesions; and the identification of the *Leishmania* species involved were recorded.

#### Group II.

This group included patients in whom a diagnosis of CL was refuted, presenting with an acute infection caused by various tropical endemic pathogens, including toxoplasmosis (with specific IgG and IgM), intestinal nematodes (*Strongyloides stercoralis* and hookworms), extraintestinal amoebiasis, *Plasmodium vivax* malaria, Chagas disease due to *Trypanosoma cruzi*, syphilis due to *Treponema pallidum*, *Mycobacterium tuberculosis* or *Mycobacterium leprae* infections, dengue, and chronic histoplasmosis.

#### Group III.

This group included patients in whom a diagnosis of CL was refuted, presenting with proven autoimmune diseases.

#### Group IV.

This control group included in- or outpatients at the dermatology department, which is the local reference ward for CL treatment, who had no past or present history of CL or other previously mentioned diseases, and who were diagnosed with a dermatological infection of viral or bacterial origin.

### Immunoblotting.

All sera were tested with the “LEISHMANIA Western blot IgG” (LDBIO Diagnostics, Lyon, France) IB, automated on an Autoblot System 20^®^ (MP Biomedicals, Santa Ana, CA), according to the manufacturer’s instructions.^18^ *Leishmania infantum* promastigote antigens were electrophoretically separated and then fixed, via electro-transfer, onto a nitrocellulose sheet cut into strips. To summarize, 25 µL of each serum sample was incubated with one nitrocellulose strip. The specific anti-*Leishmania* antibodies, potentially present in the patient’s serum, selectively bound onto the *L. infantum* antigens fixed on the strip. The strip was then incubated with alkaline phosphatase antihuman IgG conjugate, which binds to the bound anti-*Leishmania* antibodies. Finally, alkaline phosphatase enzymatic activity was revealed using the substrate. The antigens recognized by the IgG anti-*Leishmania* antibodies present in the patient’s serum sample appeared as purple transversal horizontal bands.

The *Leishmania* IB positivity criterion was the presence on the strip of at least one of the 14 kDa or 16 kDa antigenic bands (Supplemental Figure 1), which established the presence of anti-*Leishmania* IgG antibodies in the patient’s serum sample. Immunoblots were read and interpreted in duplicate by a skilled operator who was blinded regarding the patients’ diagnosis: first at the Cayenne Hospital laboratory and then at the Marseille University Hospital laboratory of parasitology–mycology. The number and the intensity of bands were recorded by each reader. No discord was found in the reading.

### Statistical analysis.

We performed two statistical analyses with and without considering patients with Chagas disease. It should be noted that Chagas and *Leishmania* serology are well known to overlap (this is mentioned in the package leaflet), which would have biased our study. We calculated the following diagnosis indices for the IB assay: sensitivity (proportion of patients with positive IB assay who actually have CL), specificity (proportion of patients with negative IB assay who actually do not have CL), positive and negative likelihood ratios, diagnostic odds ratio (DOR), Youden’s index, and the number needed to diagnose and to misdiagnose, with exact binomial 95% CIs.

## RESULTS

### Patient samples.

A single serum sample per patient, kept in the biological collection at the Cayenne Hospital laboratory (French Guiana), was tested between May 2017 and September 2018. Ultimately, 141 patients were divided into four groups.

#### Group I.

This group included 30 patients (21 men and nine women with a mean age of 33 years, ranging from 17 to 54 years) with CL (Table 1); one of whom had mucosal involvement. For three patients, the *Leishmania* species was not identified; *L. guyanensis* was involved in 23 cases and *L. braziliensis* in four. Patients presented with skin lesions spread across their lower limbs (*n* = 15), upper limbs (*n* = 13), head/neck (*n* = 6), and/or trunk (*n* = 6). In 12 patients, skin lesions were distributed across more than one body area. The mean number of lesions was two (range 1–10). The time to CL diagnosis ranged from 1 to 6 months.

**Table 1:** Clinical and demographic characteristics of patients in group I

	Demographic data		Diagnostic			Species	Evolution of symptoms (months)	Number of lesions	Location of lesions				Nature			WB result		
	Age	Gender	Microscopy	Culture	PCR				Trunk	Upper limbs	Lower limbs	Head/neck	Ulceration	Nodule	Mucosal involverment	p14	p16	Result
1	31	Male	Pos	Pos	Pos	*L. guyanensis*	3	10	1	1	8	0	Yes	Yes	No	Yes	–	Pos
2	32	Male	Pos	Uninterpretable	Pos	*L. guyanensis*	1	1	0	0	1	0	Yes	No	–	Yes	–	Pos
3	27	Male	Pos	Uninterpretable	Pos	*L. guyanensis*	–	2	0	1	0	1	–	–	–	Yes	–	Pos
4	54	Female	Neg	Pos	–	*L. guyanensis* complex	1	1	1	0	0	0	Yes	No	No	–	–	Neg
5	26	Male	Neg	Pos	–	*L. guyanensis* complex	6	5	3	2	0	0	–	–	No	Yes	Yes	Pos
6	32	Female	Pos	Uninterpretable	Pos	*L. guyanensis*	–	1	0	0	1	0	–	–	No	–	Yes	Pos
7	38	Male	Neg	Uninterpretable	Pos	*L. guyanensis*	1	1	0	0	1	0	–	–	–	Yes	–	Pos
8	25	Male	Pos	Pos	Pos	*L. guyanensis*	2	2	0	2	0	0	Yes	No	No	Yes	Yes	Pos
9	31	Female	Neg	Neg	Pos	*–*	1	2	0	0	2	0	Yes	No	–	Yes	Yes	Pos
10	39	Male	Pos	Pos	Pos	*L. guyanensis*	3	1	0	0	1	0	Yes	No	–	Yes	Yes	Pos
11	29	Male	Pos	Uninterpretable	Pos	*L. guyanensis*	–	1	0	0	0	1	Yes	No	No	Yes	Yes	Pos
12	27	Male	Neg	Pos	Pos	*L. guyanensis*	–	1	0	0	0	1	–		No	–	–	Neg
13	24	Male	Pos	Neg	–	*–*	2	1	0	1	0	0	Yes	No	No	Yes	Yes	Pos
14	31	Male	Pos	Pos	Pos	*L. guyanensis*	3	1	0	0	1	0	–	–	No	Yes	–	Pos
15	38	Male	Pos	Uninterpretable	Pos	*L. guyanensis*	1	1	0	1	0	0	–	–	No	Yes	Yes	Pos
16	28	Male	Pos	Pos	Pos	*L. guyanensis*	2	4	0	4	0	0	Yes	No	–	Yes	Yes	Pos
17	21	Male	Pos	Pos	Pos	*L. guyanensis*	2	4	0	2	2	0	Yes	No	–	Yes	Yes	Pos
18	47	Female	Pos	Pos	Pos	*L. guyanensis*	1	3	2	1	0	0	Yes	Yes	No	Yes	–	Pos
19	31	Female	Pos	Uninterpretable	Pos	*L. guyanensis*	3	4	1	0	1	2	Yes	Yes	–	Yes	Yes	Pos
20	50	Female	Neg	Pos	Pos	*–*	1,5	1	0	0	1	0	Yes	Yes	No	–	–	Neg
21	31	Female	Pos	Pos	Pos	*L. guyanensis*	3	4	1	0	1	2	Yes	Yes	–	Yes	Yes	Pos
22	33	Female	Neg	Pos	Pos	*L. guyanensis*	1,5	1	0	1	0	0	Yes	No	–	Yes	Yes	Pos
23	17	Female	Pos	Pos	Pos	*L. guyanensis*	2	2	0	0	2	0	Yes	Yes	No	Yes	Yes	Pos
24	22	Male	Pos	Pos	Pos	*L. guyanensis*	–	1	0	0	1	0	Yes	No	–	Yes	Yes	Pos
25	40	Male	Neg	Pos	Pos	*L. braziliensis*	2,5	1	0	0	0	1	Yes	No	No	Yes	–	Pos
26	30	Male	Neg	Pos	Pos	*L. braziliensis* complex	1,5	2	0	1	1	0	Yes	No	Yes	Yes	Yes	Pos
27	41	Male	Pos	Pos	–	*L. guyanensis* complex	1,5	1	0	1	0	0	Yes	No	No	Yes	Yes	Pos
28	18	Male	Pos	Pos	–	*L. braziliensis* complex	2	1	–	–	–	–	–	–	–	–	Yes	Pos
29	43	Male	Pos	Uninterpretable	Pos	*L. guyanensis*	–	1	0	1	0	0	Yes	No	No	Yes	Yes	Pos
30	45	Male	Pos	Neg	–	*L. braziliensis*	2	1	0	0	1	0	Yes	No	No	Yes	–	Pos

*L. braziliensis = Leishmania braziliensis*; *L. guyanensis = Leishmania guyanensis*; Neg = negative; Pos = positive.

#### Group II.

This group included 71 non-CL patients (40 men and 31 women with a mean age of 42 years, ranging from 12 to 90 years). Patients presented with toxoplasmosis (*n* = 11), histoplasmosis (*n* = 10), intestinal nematodes (*n* = 12), extraintestinal amoebiasis (*n* = 6), *P. vivax* malaria (*n* = 10), *T. cruzi* infection (*n* = 5), syphilis (*n* = 11), *M. tuberculosis* or *M. leprae* infection (*n* = 5), and dengue (*n* = 1).

#### Group III.

This group included 11 patients (three men and eight women with a mean age of 40 years, ranging from 20 to 67 years) with autoimmune diseases: cutaneous or systemic lupus erythematosus (*n* = 7) or autoimmune bullous diseases (*n* = 4).

#### Group IV.

This group included 29 in- or outpatients at the dermatology department (19 men and 10 women with a mean age of 53 years, ranging from 23 to 84 years) with viral or bacterial skin infections. Evaluation of the *Leishmania* Western blot test was carried out on the four patient groups.

### Leishmania immunoblot findings.

Group I: Antibodies directed against the 14 and/or 16 kDa antigens were detectable in 27 of the 30 CL patients’ sera (Table 1), yielding 90% (95% CI [74–97]) sensitivity. There was no correlation between band intensity and active disease or time to diagnosis. In the three patients with a negative IB, CL had been diagnosed by culture or PCR, and microscopic examination was negative in each of them. They presented only one lesion each. *Leishmania guyanensis* was identified in two, but the species was not identified in the last patient.

In group II, 26 patients (37%) displayed a positive *Leishmania* IB (Table 2): 36% (4/11) of patients with toxoplasmosis, 30% (3/10) of patients with histoplasmosis, 25% (3/12) of patients with intestinal nematodes, 50% (3/6) of patients with extraintestinal amoebiasis, 30% (3/10) of patients with *P. vivax* malaria, 80% (4/5) of patients with Chagas disease, 36% (4/11) of patients with syphilis, and 40% (2/5) of patients with *M. tuberculosis* or *M. leprae* infection. Sometimes, both p14 and p16 bands were present. In group III, four (36%) patients with autoimmune disease displayed a positive *Leishmania* IB. In the control group IV, nine (30%) of 30 patients displayed a positive *Leishmania* IB (Table 2).

**Table 2:** Results of p14 and/or p16 detection in groups I, II, III, and IV

Group	Pathology	Patients	p14	p16	p14 and p16	Positive	Negative
Group I	Cutaneous leishmaniasis	30	8 (27%)	2 (7%)	17 (57%)	27 (90%)	3 (10%)
Group II	Toxoplasmosis*	11	3 (27%)	0	1 (9%)	4 (36%)	7 (64%)
	Histoplasmosis†	10	0	1 (10%)	2 (20%)	3 (30%)	7 (70%)
	Intestinal nematodes‡	12	1 (8%)	1 (8%)	1 (8%)	3 (25%)	9 (75%)
	* Strongyloides stercoralis*	6	0	0	1 (17%)	1 (17%)	5 (83%)
	* Ancylostoma duodenale*/*Necator americanus*	6	1 (17%)	1 (17%)	0	2 (33%)	4 (67%)
	Positive amoebiasis serology§^‖^	6	1 (16%)	2 (33%)	0	3 (50%)	3 (50%)
	Malaria due to *Plasmodium vivax*¶	10	1 (10%)	0	2 (20%)	3 (30%)	7 (70%)
	Syphilis#	11	2 (18%)	0	2 (18%)	4 (36%)	7 (64%)
	Mycobacterium**††	5	2 (40%)	0	0	2 (40%)	3 (60%)
	Mycobacterium tuberculosis**	3	1 (33%)	0	0	1 (33%)	2 (67%)
	* Mycobacterium leprae*††	2	1 (50%)	0	0	1 (50%)	1 (50%)
	Chagas disease‡‡	5	1 (20%)	0	3 (60%)	4 (80%)	1 (20%)
	Dengue§§	1	0	0	0	0	1 (100%)
Group III	Autoimmune disease^‖‖^¶¶	11	1 (9%)	2 (18%)	1 (9%)	4 (36%)	6 (55%)
	* *Bullous pemphigoid^‖‖^	1	0	0	0	0	1 (100%)
	* *Pemphigus^‖‖^	1	0	0	0	0	1 (100%)
	* *Systemic lupus erythematosus¶¶	7	1 (14%)	2 (29%)	1 (14%)	4 (57%)	3 (43%)
Group IV	Dermatology patients	29	2 (7%)	4 (14%)	3 (10%)	9 (31%)	20 (69%)

Presented in the form of raw values (% recognition).

* Presence of both IgG and IgM anti–*Toxoplasma gondii*.

† Positive culture on blood/urine/bone marrow/bronchal washing and/or PCR.

‡ Microscopic examination of stool samples.

§ Latex test.

‖ Positive amoebiasis serology from Marseille (*n* = 8) are not included in group II.

¶ Thick and thin blood smear examination and rapid diagnosis testing.

# Serology showing VDRL > 8 and TPHA > 160.

** Diagnosis performed by culture on sputum, bronchial fluid, lymph nodes, or bone marrow.

†† Diagnosis performed by culture on skin biopsy, nasal smear, or dermal juice.

‡‡ Positive culture or PCR on blood samples.

§§ Positive antigen NS1 or PCR.

‖‖ Positive direct/indirect immunofluorescence associated with compatible symptoms.

¶¶ According to the American College of Rheumatology criteria.

Finally, IB diagnosis of New-World CL, excluding patients with Chagas disease, showed a good sensitivity of 90% and a relatively low specificity of 66% (0.62–0.68). The other diagnostic indices were calculated: positive likelihood ratio (LR+) = 2.65 (95% CI [1.93–3.05]), negative LR^−^ = 0.15 (95% CI [0.04–0.42]), DOR = 17.5 (95% CI [4.61–78.02]), Youden’s index = 0.56 (95% CI [0.36–0.65]), number needed to diagnose = 1.79 (95% CI [1.53–2.80]), and number needed to misdiagnose = 3.49 (95% CI [2.81–3.93]). Taking into account the patients with Chagas disease, the sensitivity is 90% and the specificity is 64% (0.60–0.66). The other diagnostic indices were calculated: LR+ = 2.50 (95% CI [1.84–2.86]), LR− = 0.16 (95% CI [0.04–0.43]), DOR = 16.0 (95% CI [4.24–70.83]), Youden’s index = 0.56 (95% CI [0.36–0.65]), number needed to diagnose = 1.85 (95% CI [1.58–2.96]), and number needed to misdiagnose = 3.28 (95% CI [2.68–3.65]). The 90% sensitivity indicates that a positive *Leishmania* IB is associated with a high probability of CL.

## DISCUSSION

The aim of this study was to establish the strength and limitation of IB detection of anti–*Leishmania*-specific antigens for the diagnosis of CL due to *L. guyanensis* and *L. braziliensis* in French Guiana. We hypothesized that major *L. infantum* antigens 14 and 16 kDa have interspecies specificity^13^ and, therefore, are of potential interest for the *Vianna* complex. We used the LEISHMANIA Western blot IgG (LD Bio, Lyon, France) kit because it is a mainstay confirmatory test for the diagnosis of visceral leishmaniasis in France and the only one on the market. This test uses *L. infantum* promastigote antigens; its high sensitivity in patients infected with *L. guyanensis* and *L. braziliensis* involved in New-World CL demonstrates that this IB cross-reacts with these species and, more precisely, that antibodies directed against *L. guyanensis* and *L. braziliensis* specifically bind to the p14 and p16 *L. infantum* promastigote antigens.

Among the patients in group II who were infected with various endemic pathogens, two that cross-reacted in more than 50% with the *Leishmania* IB assay were *T. cruzi* and extraintestinal amoebiasis. The cross-reactivity of Chagasic and leishmaniasic sera is well known^19^ and is presented in the manufacturer’s instructions of the IB kit tested. This is why we calculated the specificity of the test by considering patients with and without Chagas disease. But an additional study would be necessary with a larger number of Chagasic sera to establish the specificity precisely. Despite *Leishmania* and *Trypanosoma* being present in the same areas, their clinical presentations are clearly distinct, and, therefore, they cannot be considered as differential diagnoses from a clinical point of view. However, an asymptomatic stage can be observed during American trypanosomiasis. In fact, a *Leishmania* IB–positive result requires a serological investigation of Chagas disease. Inversely, it should be noted that the test cannot be used for CL diagnosis in a patient who has been diagnosed with Chagas diseases. However, the *Leishmania* IB cross-reaction with extraintestinal amoebiasis was unexpected and, to the best of our knowledge, has never been reported.^20,21^ This disease has a distinct clinical presentation and is not a differential diagnosis of CL; this cross-reactivity was unexpected because, although both are protozoa, *Entamoeba* and *Leishmania* are phylogenetically distant genera, whereas *Trypanosoma* and *Leishmania* belong to the same Euglenozoa phylum. To verify this cross-reaction, *Leishmania* IB was carried out on eight sera of patients with proven extraintestinal amoebiasis from Marseille (laboratory of Parasitology Mycology), a non-endemic region for New-World CL, and none of the tested sera will have expressed p14 and/or p16 antigens. This result refutes the hypothesis of a cross-reaction. For other infectious pathologies, the *Leishmania* IB assay positivity was around 30% as in groups III and IV (Table 2).

The presence of 36% and 31% positive *Leishmania* IB in groups III and IV, respectively, may be linked to our sampling from endemic areas of leishmaniasis.^4^ With a mean age of 46 years, patients in groups III and IV are likely to have been in contact with the *Leishmania* parasite in their lifetime. It is known that spontaneously healing^22^ or paucisymptomatic^23^ CL presentations are frequent, and a positive IB might be due to the presence of residual antibodies caused by a past or asymptomatic infection. Cross-reactivity with autoimmune disease appears unlikely but cannot be ruled out because of the relatively small sample size. We also analyzed the higher molecular weight bands, but none could discriminate between patents with active or inactive CL.

The current CL diagnostic tools have several limitations. The direct microscopic examination of smear and cultures requires expertise and lacks sensitivity.^24^ *Leishmania* culturing is labor intensive, the time to results extends to several weeks, and it is uninterpretable in cases of contamination with bacteria or yeasts. In the presence of clinically compatible lesions, in this limited laboratory diagnosis context, a negative *Leishmania* IB assay should prompt the clinician to consider differential diagnoses. When faced with cutaneous ulcers, ecthyma, Buruli ulcer, atypical mycobacteria, and squamous cell carcinoma are the main differential diagnoses. In case of mucosal involvement, an endoscopy should be considered to rule out a neoplastic origin. Other infections such as chromomycosis or lobomycosis could be suspected in case of nodules and plaques. Therefore, a negative IB should prompt clinicians to actively document a differential diagnosis, without waiting for the results of *Leishmania* cultures.

Finally, although visceral leishmaniasis has not yet been described in French Guiana, Rotureau et al.^25^ have hypothesized that *L. infantum* may have been imported to South America by immigrants from the Old World. This hypothesis warrants the implementation of epidemiological surveillance based on this *Leishmania* IB assay at the Cayenne Hospital laboratory to prospectively test each immunocompromized patient with pancytopenia and splenomegaly, a clinical presentation compatible with visceral leishmaniasis. It is possible that visceral leishmaniasis could represent a differential diagnosis of histoplasmosis, which is routinely looked for in such patients in French Guiana.

## CONCLUSION

Our results suggest that immunoblotting may be of interest in the diagnosis excluding CL, thus joining a set of arguments in the event of difficult diagnoses in endemic areas and prompting physicians to look for another diagnosis in the event of negative IB. In case of positive result, it is necessary to do a serologic investigation of Chagas disease. It remains important to develop new serological tools based on specific strains implicated, respectively, in CL, VL, and American tegumentary leishmaniasis to increase specificity.

## Supplemental figure

Supplemental materials
