# Supplementary figures and images for: Immunoblot for the Diagnosis of Cutaneous Leishmaniasis in French Guiana
Source: Am J Trop Med Hyg. 2021 May 3;104(6):2091–6. doi: 10.4269/ajtmh.19-0591 (PMC8176500; doi:10.4269/ajtmh.19-0591)

**Supplemental figure 1.**

**
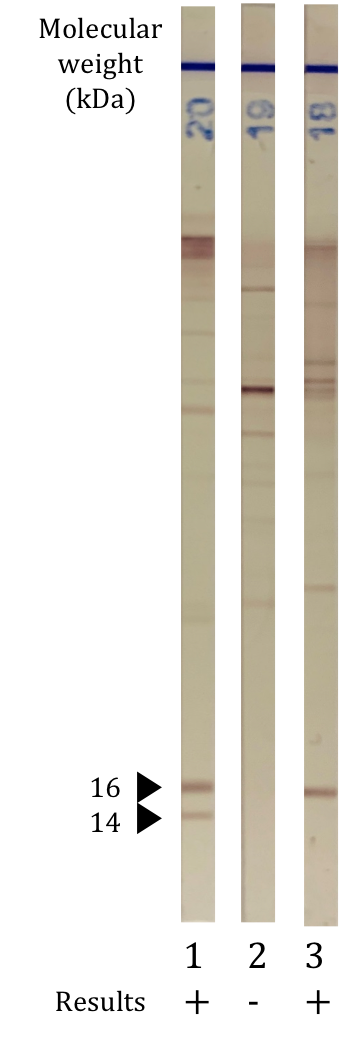
**

Supplement: Supplementary file 1 [file tpmd190591.SD1.docx]
